# *Goniothalamus flagellistylus* Tagane & V. S. Dang (Annonaceae), a new species from Mt. Hon Ba, Vietnam

**DOI:** 10.3897/phytokeys.50.4427

**Published:** 2015-05-13

**Authors:** Shuichiro Tagane, Van Son Dang, Tetsukazu Yahara, Hironori Toyama, Hop Tran

**Affiliations:** 1 Center for Asian Conservation Ecology, Kyushu University, 6-10-1 Hakozaki, Fukuoka, 812-8581, Japan; 2 The VNM Herbarium, Institute of Tropical Biology, VAST, 85 Tran Quoc Toan Street, District 3, Ho Chi Minh City, Viet Nam; 3 University of Science Ho Chi Minh City, 227 Nguyen Van Cu Street, District 5, Ho Chi Minh City, Viet Nam

**Keywords:** Annonaceae, *Goniothalamus*, Hon Ba Nature Reserve, Vietnam

## Abstract

A new species, *Goniothalamus flagellistylus* Tagane & V. S. Dang, **sp. nov.** from Hon Ba Nature Reserve in Khanh Hoa Province, South Vietnam is described and illustrated. This species is most similar to *Goniothalamus tortilipetalus* M.R.Hend., but distinct in having 308–336 stamens (vs. ca. 170–260) and ca.120 carpels (vs. ca. 50–100) per flower, and Stigma and pseudostyles ca.8.5 mm (vs. 4–4.5 mm) long.

## Introduction

The genus *Goniothalamus* (Blume) Hook. f. & Thomson, with more than 130 species (Nakkhuntod et al. 2009, Tang et al. 2013), is one of the largest genera in the Annonaceae family. The species is characterized by mostly solitary, axillary and pendent flowers, two whorls of petals with inner petals smaller than the outer ones, the inner petals connivent and forming a distinctive dome over the stamens and carpels acting as a pollination chamber and stamens having apical connectives. Most species of *Goniothalamus* are distributed in lowland evergreen rain forests in Southeast Asia, extending from India to Australia, also in New Caledonia (Jessup 1986, Mat-Salleh 2001, Saunders 2002, Saunders 2003, Kundu 2006, Saunders and Munzinger 2007, Saunders and Chalermglin 2008, Turner and Saunders 2008). In Vietnam, the following 19 species of *Goniothalamus* are recorded: *Goniothalamus chartaceus* H.L.Li, *Goniothalamus chinensis* Merr. & Chun, *Goniothalamus donnaiensis* Finet & Gagnep., *Goniothalamus elegans* Ast, *Goniothalamus expansus* Craib, *Goniothalamus gabriacianus* (Baill.) Ast (Synonym, *Goniothalamus saigonensis* Pierre ex Finet & Gagnep.), *Goniothalamus gracilipes* Bân, *Goniothalamus laoticus* (Finet & Gagnep.) Bân, *Goniothalamus macrocalyx* Bân, *Goniothalamus multiovulatus* Ast, *Goniothalamus ninhianus* Bân, *Goniothalamus takhtajanii* Bân, *Goniothalamus tamirensis* Pierre ex Finet & Gagnep., *Goniothalamus tenuifolius* King, *Goniothalamus touranensis* Ast, *Goniothalamus undulatus* Ridl., *Goniothalamus vietnamensis* Bân, *Goniothalamus wightii* Hook.f. & Thomson and *Goniothalamus yunnanensis* W.T.Wang (Finet and Gagnepain 1907, Hô 1999, Bân 2000).

During the botanical survey of Hon Ba Nature Reserve in South Vietnam, we encountered an undescribed species of *Goniothalamus* in Mt. Hon Ba at 400 m elevation. We here describe and illustrate this new species, *Goniothalamus flagellistylus* Tagane & V. S. Dang, sp. nov.

## Materials and methods

### Morphological observations

In order to verify the validity of this new species we undertook a thorough literature review, consulted specimens from the following herbaria FU, BKF, KYO, MBK and VNM, as well as online digitized plant specimens (e.g. JSTOR Global Plants).

The thickness of leaves, sepals and petals was measured using a digital caliper (Absolute Digimatic 547-401, Mitutoyo, Japan, resolution 0.001 mm).

### DNA barcoding

For DNA isolation, leaf material was collected and desiccated using silica gel in the field. DNA was extracted using a modified CTAB method in which silica-dried leaves were ground in a TissueLyser (QIAGEN), and the powder washed five times with 1 mL buffer (0.1 M HEPES, pH 8.0; 2% mercaptoetanol; 1% PVP; 0.05 M ascorbic acid) before DNA extraction. We sequenced the partial genes for the large subunit ribulose-1,5-bisphosphate carboxylase oxygenase (*rbcL*) and maturase K (*matK*), following published protocols (Kress et al. 2009; Dunning and Savolainen 2010).

## Taxonomy

### Goniothalamus flagellistylus

Taxon classificationPlantaeMagnolialesAnnonaceae

Tagane & V. S. Dang sp. nov.

urn:lsid:ipni.org:names:77147106-1

Figs 1 , 2

#### Diagnosis.

Similar to *Goniothalamus tortilipetalus* M.R.Hend., but differing from that species in having 308–336 stamens (vs. ca. 170–260) and ca.120 carpels (vs. ca. 50–100) per flower, and stigmas and pseudostyles ca.8.5 mm (vs. 4–4.5 mm) long.

#### Type.

Vietnam, Khanh Hoa Province, Hon Ba Nature Reserve, in evergreen forest near stream, 12°06.51'N, 108°59.23'E, alt. 400 m, *Tagane S., Kanemitsu H., Dang V.S., Tran H. with Hanh N., Loi X.N., Thach N.D., Dinh N., Hieu P.N.H. V1497*, 12 July 2014, Fl., holotype: KYO!; isotypes: BKF!, FU!, K!, VNM!, the herbarium of Hon Ba Nature Reserve!).

#### Description.

Small trees, 11 m tall, DBH 8 cm. Young twigs sparsely covered with brown hairs, soon glabrous, blackish when dry. Petioles 1–1.2(–1.5) cm long, 2.5–3.5 mm in diam., glabrous, black when dry. Leaf blades narrowly oblong-elliptic, 31–45 × 8.2–11.5 cm, length/width ratio 2.7–4.0, 185–225 µm thick, base acute to obtuse, margin entire, revolute when dry, apex acuminate, acumen ca. 1.5 cm long, leathery, slightly shiny above, glabrous on both surfaces; midribs impressed above, prominent below, glabrous on both surfaces, secondary veins 16–20 pairs, arising at an angle of 60–70 degrees from a midrib, prominent on both surface when dry, glabrous on both surfaces, tertiary veins distinct above, slightly distinct below. Flowers solitary, arising from main trunks and older branches, pendent; pedicels 19–25 mm long, 1.5–2.5 mm in diam., glabrous; bracts 3–4, very broadly triangular to hemiorbicular, ca. 1.6 mm long, brownish pubescent outside, glabrous inside, margin sometimes ciliate. Sepals ovate-triangular, 2.2–2.8 × 2.2–2.8 cm in flower, accrescent, increasing to 3.3 × 3.8 cm in fruit, 200–210 µm thick, basally connate, greenish in vivo, glabrous outside, sparsely covered with brown hairs inside, veins reticulated, distinct outside, indistinct inside. Outer petals oblong-ovate to narrowly ovate, 6–9. 2 × 2.2–3.1 cm, length/width ratio 2.7–3.4, 380–450 µm thick, greenish, glabrescent outside, sparsely covered with short brown hairs inside, except at base velutinous, veins faintly visible outside, indistinct inside. Inner petals rhombic, 1.6 × 0.7 cm, length/width ratio ca. 1.9, 1100–2100 µm thick, greenish, pubescent outside, velutinous inside with 12–14 basal grooves. Stamens 308–336 per flower, flattened-oblong, 3.8–4.8 × 0.6 mm, glabrous; connectives long-apiculate, 1.2–1.5 mm long, apiculate length 0.5–0.9 mm long, densely covered with cream-white hairs. Carpels ca. 120 per flower; ovary 1.4–1.7 × ca. 0.3 mm, densely covered with golden-brown hairs; stigmas and pseudostyles flagellate, ca.8.5 mm long, L-shaped curved in the middle, yellowish in vivo, blackish when dry, glabrous, tip awl-shaped. Fruits with persistent calyx, fruiting pedicels 2.7 cm long, 3–4.5 mm in diam. Monocarps 22, ellipsoid, 1.6–1.7 cm long, ca. 1.0 cm in diam., base attenuate, apex apiculate, glabrous, reddish-brown, pericarp ca. 0.5 mm thick when dry; stipes 0.6–1.2 mm long, ca. 2 mm in diam., glabrous. Seeds one per monocarp, 1.5 cm long, 0.8–1.0 cm in diam., yellowish brown, glabrous, seeds with copious surrounding mucilage.

**Figure 1.**
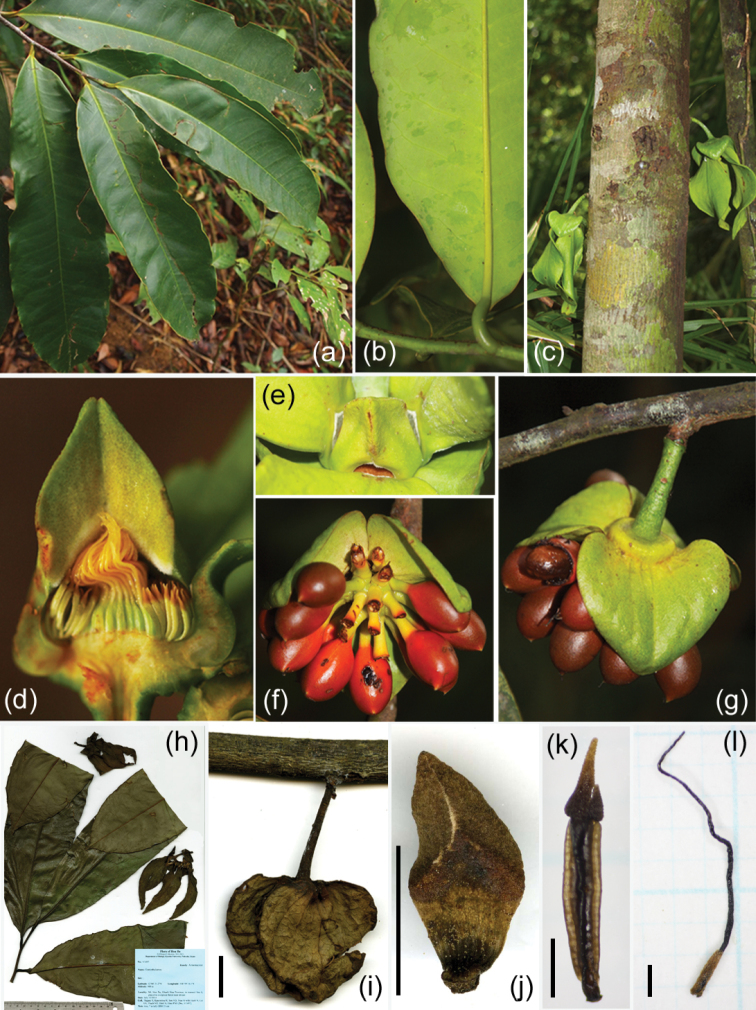
*Goniothalamus flagellistylus* sp. nov. (**a**) Leafy branch, (**b**) portion of abaxial leaf surface, (**c**) flowers on main trunk, (**d**) vertical section of flowers, (**e**) apertures between inner petals, (**f–g**) mature fruit on older blanch, (**h**) holotype, (**i**) pedicel and sepals on old branch, (**j**) adaxial side of inner petal, (**k**) stamen, (**l**) carpel. (**h–l**) From *Tagane et al. 1497*. Scale bars (**i**, **j**) = 1 cm, (**k, l**) = 1 mm.

**Figure 2.**
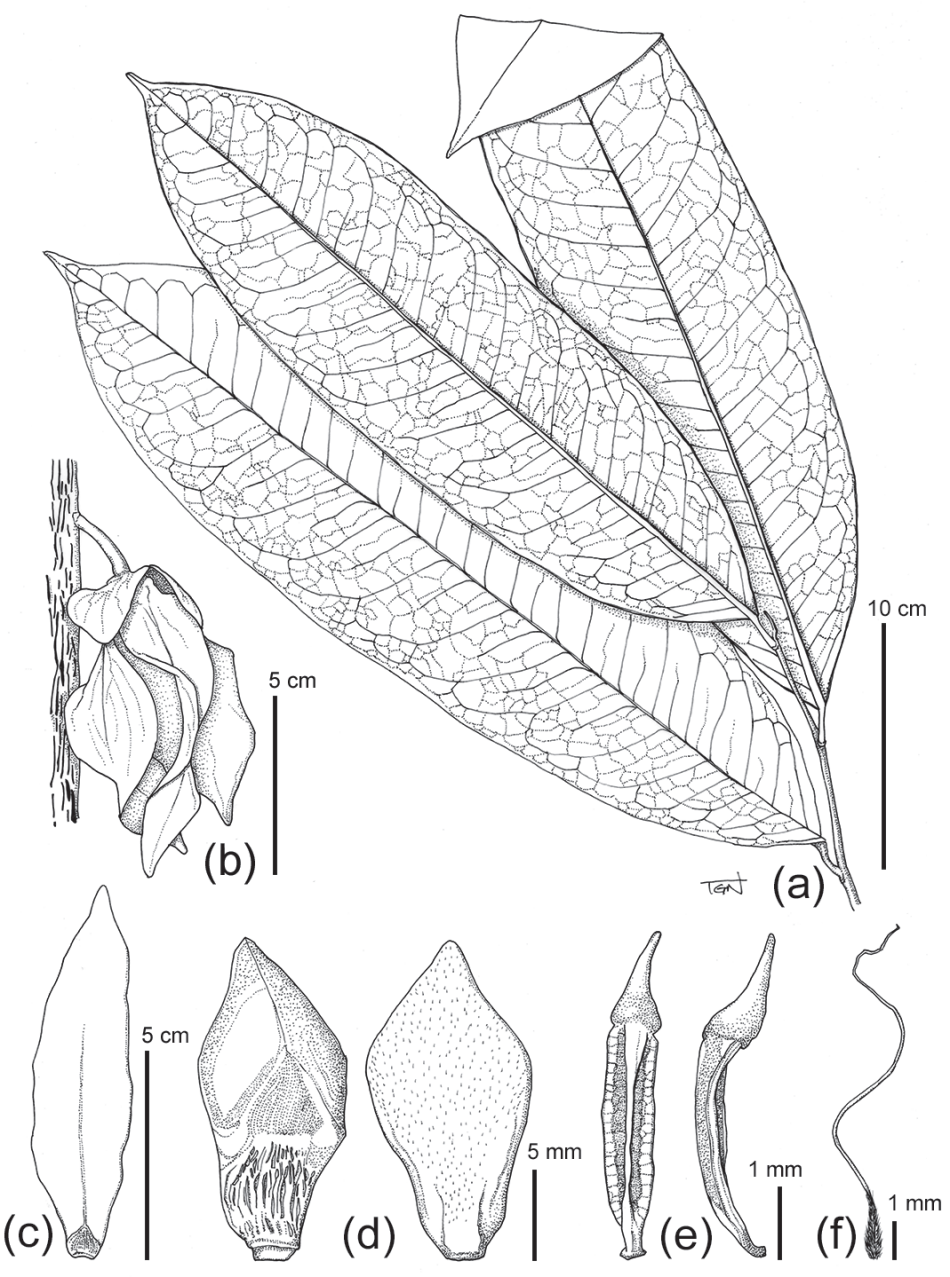
Line drawing of *Goniothalamus flagellistylus* sp. nov. (**a**) leafy twig, (**b**) flower on main trunk, (**c**) outer petal (adaxial), (**d**) inner petals (ad- and abaxial), (**e**) Stamens, (**f**) Carpel. Materials from *Tagane et al. V1497*.

#### Phenology.

Mature flowers and fruits were collected in July and November, respectively.

#### Distribution and habitat.

This species is known only from Mt. Hon Ba, Khanh Hoa Province, South Vietnam. The small population was found on a slope in broad-leaved evergreen forest, ca. 100 m apart from a rapid river, where *Ixonanthes reticulata* Jack, *Palaquium* sp., *Gironniera subaequalis* Planch., *Archidendron chevalieri* (Kosterm.) I.C.Nielsen, *Barringtonia augusta* Kurz, *Barringtonia macrostachya* (Jack) Kurz, *Camellia krempfii* (Gagn.) Sealy, *Streblus indicus* (Bureau) Corner, *Xerospermum noronhianum* Blume and *Pandanus fibrosus* Gagnep. are dominated.

#### Other specimen examined.

Vietnam, Khanh Hoa Province, Hon Ba Nature Reserve, in evergreen forest near stream, 12°06.51'N, 108°59.23'E, alt. 400 m, *Toyama H., Tagane S., Dang V.S., Nagamasu H., Naiki A., Tran H., Yang C.J. with Cuong N.Q., Hieu H.N.P. V1972*, 22 November 2014, Fr. (FU!, KYO!, NTU!, VNM!, the herbarium of Hon Ba Nature Reserve!).

#### Etymology.

The specific epithet is in reference to its flexuous styles which is too long to insert straight in the pollination chamber that formed by the inner petals.

#### GeneBank accession No.

*Tagane et al. V1497*: LC010815 (*rbcL*), LC010816 (*matK*).

#### Similar species.

*Goniothalamus flagellistylus* is morphologically similar to *Goniothalamus calvicarpus* Craib, *Goniothalamus griffithii* Hook.f. & Thomson, and *Goniothalamus tortilipetalus*, all of which form a monophyletic group (Nakkuntod et al. 2009) and are characterized by relatively large sepals with distinct veins and stamens with long apiculate connectives (Saunders and Chalermglin 2008). Among the three species, *Goniothalamus flagellistylus* is most similar to *Goniothalamus tortilipetalus*, which is distributed in the Malay Peninsula, in having more than 200 stamens and more than 50 carpels. In fact, The BLAST similarity search based on the *matK* sequence of *Goniothalamus flagellistylus* resulted in homology as high as 734/736 bp with the sequence of *Goniothalamus tortilipetalus* (GeneBank accession no. EU715081) in the DNA database. However, as described in the diagnosis above, the two species can be easily distinguished by the number of stamens and carpels per flower, and style length (Table 1). Also, *Goniothalamus flagellistylus* is different from *Goniothalamus tortilipetalus* in that flowers arise not only from the main trunk but also from the older branches as in *Goniothalamus calvicarpus* and *Goniothalamus griffithii* (Saunders and Chalermglin 2008).

**Table 1.** Morphological comparison between *Goniothalamus flagellistylus* sp. nov. and *Goniothalamus tortilipetalus* (modified from Henderson 1933; Saunders 2003; Saunders and Chalermglin 2008).

Characters	*Goniothalamus flagellistylus*	*Goniothalamus tortilipetalus*
Flowers position	On main trunk and older branches	On main trunk only
Pedicel length	19–25 mm	20–37 mm
Sepals in flowering	22–28 by 22–28 mm	19–31 by 15–26 mm
Outer petals	60–92 by 22–31 mm	35–100 by 10–25 mm
Inner petal length/width ratio	1.9	2.2–3.1
Stamen number per flower	308–336	~170–260
Carpel number per flower	120	~50–100
Stigmas and pseudostyles	ca. 8.5 mm long	4–4.5 mm long
Ovary indument	densely hairy	(Very) densely hairy

#### Conservation status.

Data deficient. *Goniothalamus flagellistylus* is known from a single population, including only six individuals: only one produces flowers/fruits while the others are just saplings. This situation satisfies the CR (critically endangered) status in criterion D of IUCN Red List Categories (IUCN 2014). However, more individuals could be found if neighboring areas are more thoroughly surveyed. Thus, we regard the conservation status as DD. In Mt. Hon Ba, large areas of primary evergreen forest below 300–400 m elevation had been cleared or selectively logged before the Hon Ba Nature Reserve established, and it is likely that some habitats of this species were lost. The forest habitat where we found *Goniothalamus flagellistylus* remains less disturbed under a lower level of logging activities probably because of its landform of the steep slope near the rapid river. The forest in this Nature Reserve is currently protected well from anthropogenic activities, and recovering better and better. The current data available are not enough for a risk evaluation, we therefore need special attention to the individuals/populations of *Goniothalamus flagellistylus* and its distribution.

## Supplementary Material

XML Treatment for Goniothalamus flagellistylus

## References

[B1] Bân NT (2000) Thực vật chí Việt Nam, Vol. 1 Science and Technics Publishers, Hanoi.

[B2] Dunning LT Savolainen V (2010) Broad-scale amplification of matK for DNA barcoding plants, a technical note. Botanical Journal of the Linnean Society 164: 1–9. doi: 10.1111/j.1095-8339.2010.01071.x

[B3] Finet AE Gagnepain F (1907) *Goniothalamus*. In: Lecomte H Gagnepain F (Eds) Flore générale de l’Indo-Chine. Masson, Paris, 1, 86–90.

[B4] Henderson MR (1927) Additions to the flora of the Malay Peninsula. Gardens’ Bulletin, Straits Settlements, Series 3(7): 87–127.

[B5] Hô PH (1999) Cay Co Viet Nam: An Illustrated Flora of Vietnam Vol. 1 Published by the author, Montreal.

[B6] IUCN (2014) The IUCN Red List of Threatened Species. Version 2014.3. http://www.iucnredlist.org [accessed 30.01.2015]

[B7] Jessup LW (1986) The genus *Goniothalamus* (Blume) JD Hook. & Thomson (Annonaceae) in Australia. Austrobaileya 2(3): 224–226.

[B8] Kress WJ Erickson DL Jones FA Swenson NG Perez R Sanjur O Bermingham E (2009) Plant DNA barcodes and a community phylogeny of a tropical forest dynamics plot in Panama. Proceedings of the National Academy of Sciences of the United States of America 106(44): 18621–18626. doi: 10.1073/pnas.0909820106 19841276 10.1073/pnas.0909820106 PMC2763884

[B9] Kundu SR (2006) A synopsis of Annonaceae in Indian subcontinent: Its distribution and endemism. Thaiszia Journal of Botany 16: 63–85.

[B10] Mat-Salleh K (2001) New and noteworthy species of Bornean *Goniothalamus* (Annonaceae). Folia Malaysiana 2: 75–116.

[B11] Nakkuntod M Su YC Seelanan T Saunders RMK (2009) Molecular phylogenetic and morphological evidence for the congeneric status of *Goniothalamus* and *Richella* (Annonaceae). Taxon 58(1): 127–132.

[B12] Saunders RMK (2002) The genus *Goniothalamus* (Annonaceae) in Sumatra. Botanical Journal of the Linnean Society 139(3): 225–254. doi: 10.1046/j.1095-8339.2002.00061.x

[B13] Saunders RMK (2003) A synopsis of *Goniothalamus* species (Annonaceae) in Peninsular Malaysia, with a description of a new species. Botanical Journal of the Linnean Society 142(3): 321–339. doi: 10.1046/j.1095-8339.2003.00177.x

[B14] Saunders RMK Chalermglin P (2008) A synopsis of *Goniothalamus* species (Annonaceae) in Thailand, with descriptions of three new species. Botanical Journal of the Linnean Society 156(3): 355–384. doi: 10.1111/j.1095-8339.2007.00762.x

[B15] Saunders RMK Munzinger J (2007) A new species of *Goniothalamus* (Annonaceae) from New Caledonia, representing a significant range extension for the genus. Botanical Journal of the Linnean Society 155(4): 497–503. doi: 10.1111/j.1095-8339.2007.00718.x

[B16] Tang CC Xue B Saunders RMK (2013) A new species of *Goniothalamus* (Annonaceae) from Palawan, and a new nomenclatrual combination in the genus from Fiji. Phytokes 32: 27–35. doi: 10.3897/phytokeys.32.6663 10.3897/phytokeys.32.6663 PMC3881350 24399904

[B17] Turner I Saunders RMK (2008) Four new species of *Goniothalamus* (Annonaceae) from Borneo. Nordic Journal of Botany 26: 329–337. doi: 10.1111/j.1756-1051.2008.00359.x

